# Supplementary material for: Advancements in Telomerase-Targeted Therapies for Glioblastoma: A Systematic Review
Source: Int J Mol Sci. 2024 Aug 9;25(16):8700. doi: 10.3390/ijms25168700 (PMC11354571; doi:10.3390/ijms25168700)
Supplement: Supplementary file 1 [file ijms-25-08700-s001.zip › ijms-3099171-supplementary.pdf]

Supplementary Material S1 - Table S1: Features of the model system used for testing the hTERT inhibition molecules

Author	*Drug*	*Cell Lines*	*Features of model system*						
			RT resistant	*PTEN-WT*	*SCID Model*	*RT Sensitive*	*DNA-PKcs*	*GBM cell lines*	*Xenograft*
Mirzazadeh et al.[14]	Resveratrol (RSV)	U87MG						✓	
Gurung et al. [15]	Thymoquinone (TQ)	M059K					Normal	✓	
		M059J					Down	✓	
Khaw et al[16- 19]	Curcumin, Plumbagin, Genistein, TSA	KNS60	✓					✓	
		U251MG	✓					✓	
		A172				✓		✓	
		ONS76	✓						
Lin et al [20]	Butylidenephthalide (BP)	DBTRG-05MG						✓	✓
		GBM 8401						✓	
Kiaris and Schally et al [21]	MZ-5-156 (GH-RH antagonist)	U87MG						✓	✓ (athymic nude)
Udroiu et al [22]	Epigallocatechingallate (EGCG)	U251MG	✓						
Das et al [23]	Retinoids	C6 rat glioma cells						✓	
Aquilanti et al [5]	CRISPRi approach	LN18							
		98G			✓			✓	✓
		SF295							
Ergüven et al [24]	Suramin	C6 glioma cell line						✓	✓ (rat Wistar)
Lavanya et al. [26]	BIBR1532	LN18						✓	
Biray Avci et al [28]	BIBR1532	U87MG						✓	
Ahmad et al [29]	Costunolide	A172						✓	
		U87MG						✓	✓
		T98G						✓	
Gurung et al [30]	MST-312	M059K					Normal	✓	
		M059J					Down	✓	
		KNS60	✓					✓	
		ONS76	✓					✓	

Takahashi et al [32]	Eribulin	U87MG						✓	
		U118MG						✓	
		U251MG						✓	✓ (nude mice)
		LN229						✓	
Cheng et al [33]	Arsenico	U87						✓	
		U251						✓	
		SHG44						✓	
		C6						✓	
Marian et al [38]	Imetelstat	Tumors samples						✓	✓ (Orthotopic)
Ferrandon et al [39]	Imetelstat	U87MG						✓	✓ (Orthotopic)
Ozawa et al [40]	GRN163	U251						✓	✓ (athymic rat)
		U87						✓	✓
Hashizume et al [41]	GRN163	U-251 MG						✓	✓ (athymic rat)
Bejarano et al [43]	TRF1 Inhibition	U251						✓	✓ Syngeneic mice
		U87							
		T98G							
		293T							
		DF1							
Zhou et al [44]	BRACO-19	U87						✓	
		U251							
		SHG-44							
		C6 rat							
Lagah et al [45]	RHPS4	U87 MG							✓
Berardinelli et al [46]	RHPS4	U251MG						✓	
		U87MG							
Hasegawa et al [47]	Telomestatin	GBM146						✓	
		GBM157							
Merle et al [48]	N-methylated triflate (TAC)	SF763	✓					✓	
		SF767	✓					✓	
Mancini et al [55]	siRNA GABPβ1L inhibition	GBM1						✓	✓(Athymic mice)
		T98G							
		LN229							

George et al [52]	hTERT siRNA + IFN-γ	LN18		✓				✓	✓
		SNB19						✓	
Falchetti et al [53,54]	hTERT siRNA	TB10						✓	✓
		U87MG						✓	
Lavanya et al [26]	hTERT siRNA	LN18						✓	
Wang et al [56]	Anti-miR21	U87						✓	✓ (nude mice)
		LN229						✓	
Kim et al [57]	shMUC1	U373						✓	
		T98G							
		A172							
Vinchure et al [58]	miR-490	U87MG						✓	
		T98G							

.
